# 

**Published:** 1863-04

**Authors:** 


### BOOKS AND PAMPHLETS RECEIVED.

*Transactions of the Seventeenth Annual Meeting of the Ohio State Medical Society, held at Ohio White Sulphur Springs, June* 17*th and* 18*th*, 1862.

*Biennial Report of the Board of Trustees of the Michigan Asylum for the Insane, for the years* 1861–2.

*Complete Record of the Surgery of the Battles fought near Vicksburg, December* 27, 28, 29 and 30, 1862. By E. Andrews, *late Surgeon of the First Regiment Illinois Light Artillery, and Professor of Surgery in the Medical Department of Lind University.*

*The Heritage of Mankind; or, Common Sense the Arbiter of the Medical Profession; by* Wilburn Whitley, M. D., *Physician and Surgeon, Conneautville, Penn.*

*Sanitary Commission, No.* 57—*Reports of the operations of the Inspectors and Relief Agents of the Sanitary Commission, after the Battle of Fredericksburg, December* 13, 1862, *by* J. H. Douglas, M. D., *Associate Secretary San. Com., and* C. W. Brink, M. D., *Inspector Sanitary Commission.*

*Significance of a Diploma—a Valedictory Address delivered before the Graduating Class of the Berkshire Medical College, November* 19*th*, 1862, *by* Wm. Warren Greene, M. D.

*Thirty-Ninth Annual Register of the Rensselaer Polytechnic Institute, Troy, N. Y., for the Academic Year*, 1862–63.

*Hints on the Treatment of Strangulated Hernia; the Properties of Opium as Antiphlogistic, Anatomically and Physiologically Explained, by* John O'Reilly, M. D., F. R. C. S. C., *read before the Academy of Medicine of New York. New York: Published by* William Wood, 61 *Walker Street*, 1863.

*Catalogue of Medical, Surgical, Dental and Scientific Books. By* Lindsay & Blakiston, *of Philadelphia.*

We have received this Catalogue containing an account of a very full assortment of works in every department of *Medicine, Surgery* and the Collateral Sciences. The prices are also appended to the account of each book, and they are sent by mail, free of postage, upon receipt of the price as annexed.

